# Avoiding Femoral Tunnel Convergence and Graft Damage While Performing Anterior Cruciate Ligament Reconstruction With Lateral Extra‐articular Tenodesis

**DOI:** 10.1002/atn2.70178

**Published:** 2026-07-07

**Authors:** Matthew Frederickson, Shannon Tse, Abby Viglione, Cassandra A. Lee, Ryan D. Freshman

**Affiliations:** ^1^ Department of Orthopaedic Surgery University of California Davis Sacramento California U.S.A.

## Abstract

Lateral extra‐articular tenodesis (LET) procedures have become increasingly popular in recent years as adjuncts to anterior cruciate ligament (ACL) reconstruction. One potential complication of combining LET with ACL reconstruction is tunnel convergence, which can lead to inadvertent loss of femoral ACL graft fixation. While modern LET techniques have incorporated suture anchors to avoid tunnel convergence, there is still a significant risk of anchor‐tunnel convergence leading to loss of ACL graft fixation, especially when utilizing suspensory fixation devices and anteromedial portal femoral drilling. Previous techniques have described drill angulation and high knee flexion angles during LET tunnel/anchor placement to avoid this complication but are highly dependent upon user experience or may require additional operating room assistants for leg positioning. We describe our surgical technique to avoid damaging ACL graft suspensory fixation devices when performing ACL reconstruction with a concurrent modified Lemaire LET.

## Graphical abstract

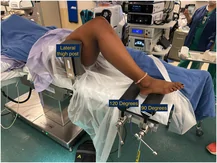

**VIDEO 1:** Surgical technique for anterior cruciate ligament reconstruction with modified Lemaire lateral extra‐articular tenodesis showing a method to avoid femoral tunnel convergence and graft damage. The patient is positioned supine with the multiple foot posts for variable flexion positioning. Key steps shown include the following: (1) lateral incision and harvest of posterior iliotibial band strip with preservation of its distal attachment to Gerdy's tubercle, (2) preparation of the iliotibial band with proximal whipstitch sutures, (3) passage of the graft deep to the lateral collateral ligament, (4) arthroscopic creation of the femoral anterior cruciate ligament tunnel using a flexible guide pin system, (5) lateral extra‐articular tenodesis anchor pilot hole drilling with simultaneous intra‐articular visualization through the anteromedial portal to confirm safe drill trajectory and avoid femoral tunnel breach, and (6) final graft tensioning and fixation at 30° of knee flexion with the tibia in neutral rotation. This technique provides both visual and tactile feedback to identify potential tunnel convergence, eliminating the need for knee hyperflexion while protecting the anterior cruciate ligament graft and suspensory fixation. Video content can be viewed at https://doi.org/10.1002/atn2.70178.

Lateral extra‐articular augmentation procedures for anterior cruciate ligament reconstructions (ACLRs) have become increasingly popular in recent years, with multiple studies showing their benefits in both primary and revision ACLRs.^1^, ^2^ By reducing anterolateral rotational laxity, lateral extra‐articular augmentation procedures can improve knee stability, lower graft failure rates, and improve patient‐reported outcome measures without negatively impacting complication or return‐to‐sport rates compared with isolated ACLR.^1^, ^3^

Various methods of lateral augmentation have been described, including modifications of the Lemaire lateral extra‐articular tenodesis (LET) and anterolateral ligament reconstruction (ALLR) techniques. These methods comparably and effectively reinforce and reduce strain on the ACL graft, yet they are not without challenges. One of the most significant issues is tunnel convergence, a phenomenon where the femoral and tibial bone tunnels created for ACLR and LET converge or overlap. Tunnel convergence has been reported at rates as high as 70% in cadaveric studies and poses risks of inadvertent early graft damage and compromised fixation.^4^, ^5^ Strategies described to mitigate this include choosing a proximal and anterior drill angulation or drilling at 130° to 140° of knee hyperflexion.^6^ Other preferred approaches include outside‐in ACL femoral drilling and cortical fixation techniques.^7^ All‐suture anchors have also been utilized as they can be placed through a smaller pilot hole, allowing surgeons to avoid drilling a full tunnel altogether when performing an LET.^8^

Although all‐suture anchors avoid the need to drill a true tunnel, many techniques describe drilling tunnels and passing the ACL graft prior to insertion of the anchor. This sequence creates a unique concern when utilizing suspensory devices for femoral ACL fixation, as both the drill trajectory for the LET and the sharp prongs on anchor inserters can converge with the femoral tunnel. Even staple fixation, which also avoids drilling a full tunnel, has been shown to violate the ACL femoral tunnel in up to 40% of cases.^9^ This risks direct damage to the graft and/or suspensory suture devices with the potential to partially or completely compromise ACL graft fixation. Conversely, if the LET anchor is placed before drilling the femoral tunnel, there is a risk of damaging the anchor during creation of the tunnel. Intraoperative arthroscopic visualization of the femoral tunnel during LET drill has been proposed to identify convergence in real time, allowing for correction of drill trajectory before damage occurs.^10^, ^11^ Although this approach addresses the limitations of relying solely on anatomic landmarks, it provides only visual feedback. We describe a surgical technique that builds upon this concept by utilizing a flexible femoral guide pin to provide both tactile and visual feedback during modified Lemaire LET anchor placement, eliminating the need for knee hyperflexion while avoiding damage to ACL graft suspensory fixation devices.

## SURGICAL TECHNIQUE

This technical note describes a method of avoiding damage to ACL graft suspensory fixation devices when performing a modified Lemaire LET using a knotless all‐suture anchor. The technique uses a flexible femoral guide pin to (1) avoid the need to maintain knee hyperflexion and (2) provide tactile feedback in addition to visual feedback to identify proper trajectory for the suture anchor pilot hole. The pearls and pitfalls (Table 1) of this procedure, as well as the advantages and disadvantages (Table 2), are described below. The technique is shown in detail in Video 1.

**TABLE 1:** Pearls and Pitfalls

**Pearls**	**Pitfalls**
Medial portal must be large enough to simultaneously accommodate flexible guide pin and camera	Improper LET anchor placement may lead to poor graft isometry and early failure
Cycle drill to clear sufficient bone from pilot hole prior to anchor insertion	LET anchor deployment within femoral tunnel may prevent proper ACL graft seating
Use of all‐suture anchor avoids hardware impingement into femoral tunnel	
LET graft length must be sufficient enough to double back over anchor	

ACL, anterior cruciate ligament; LET, lateral extra‐articular tenodesis.

**TABLE 2:** Advantages and Disadvantages

**Advantages**
Combined visual and tactile feedback to avoid LET anchor convergence with femoral tunnel
Flexible guide pin avoids the need for assistant to maintain hyperflexion while placing LET anchor and allows for simultaneous placement of guide pin and arthroscope through medial portal
Early placement of all‐suture LET anchor prevents inadvertent disruption of tightrope suspensory devices after graft fixation due to sharp LET anchor insertion prongs

**Disadvantages**
Disrupts normal flow of ACL reconstruction procedure by requiring additional transition from intra‐articular to extra‐articular work
Use of flexible guide pin requires specialized guides and flexible reamers

ACL, anterior cruciate ligament; LET, lateral extra‐articular tenodesis.

### Patient Positioning

**FIGURE 1:**
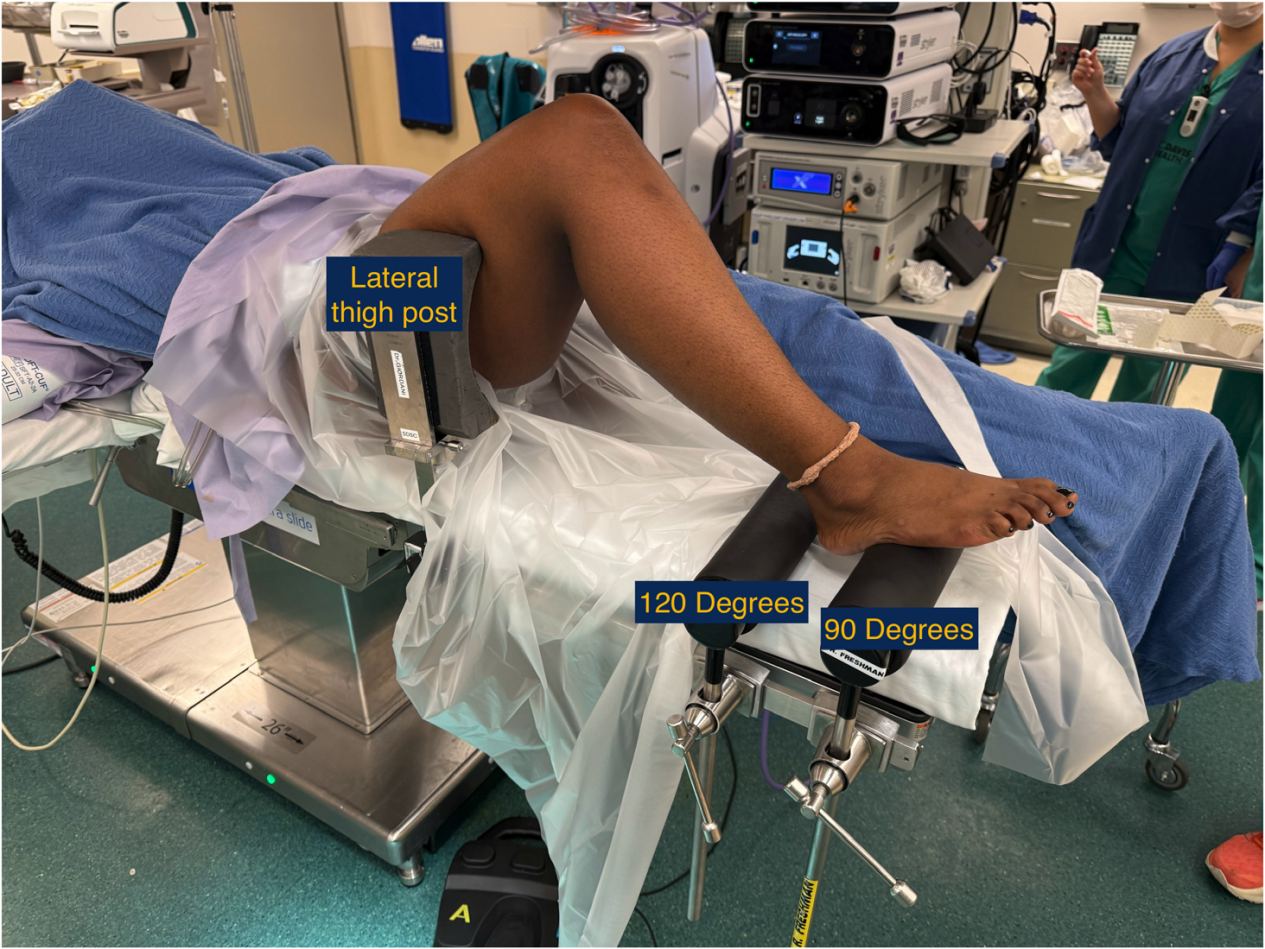
The right knee is shown in supine position with the knee flexed to approximately 90°. Patient positioning and operative setup for anterior cruciate ligament reconstruction with LET. The patient is positioned supine with the operative knee supported by dual foot positioners, allowing positioning at approximately 90° and 120° of knee flexion as needed during different stages of the procedure. A lateral thigh post is placed proximal to the knee to permit controlled valgus stress during arthroscopy. The lateral aspect of the knee is fully exposed and prepped to allow seamless transition between intra‐articular arthroscopy and open lateral access for LET graft harvest and fixation without repositioning the patient. (LET, lateral extra‐articular tenodesis.)

The patient is positioned supine on the operating table, and a nonsterile thigh tourniquet is applied. A collapsible lateral C‐post is placed at the level of the tourniquet, and multiple foot posts are used at the end of the table to attain 90° and 120° of knee flexion (Figure 1). The operative leg is prepared and draped according to the surgeon's preferred method. Anatomic landmarks, including Gerdy's tubercle, the lateral epicondyle, and the femoral shaft are palpated.

### Equipment

The following equipment was used: FlipCutter Flexible Reaming System including flexible guide pin, flexible reamer, and femoral aiming guide (Stryker, Kalamazoo, MI, USA) and 2.6 mm Knotless FiberTak All‐Suture Anchor (Arthrex, Naples, FL, USA).

### Surgical Technique

**FIGURE 2:**
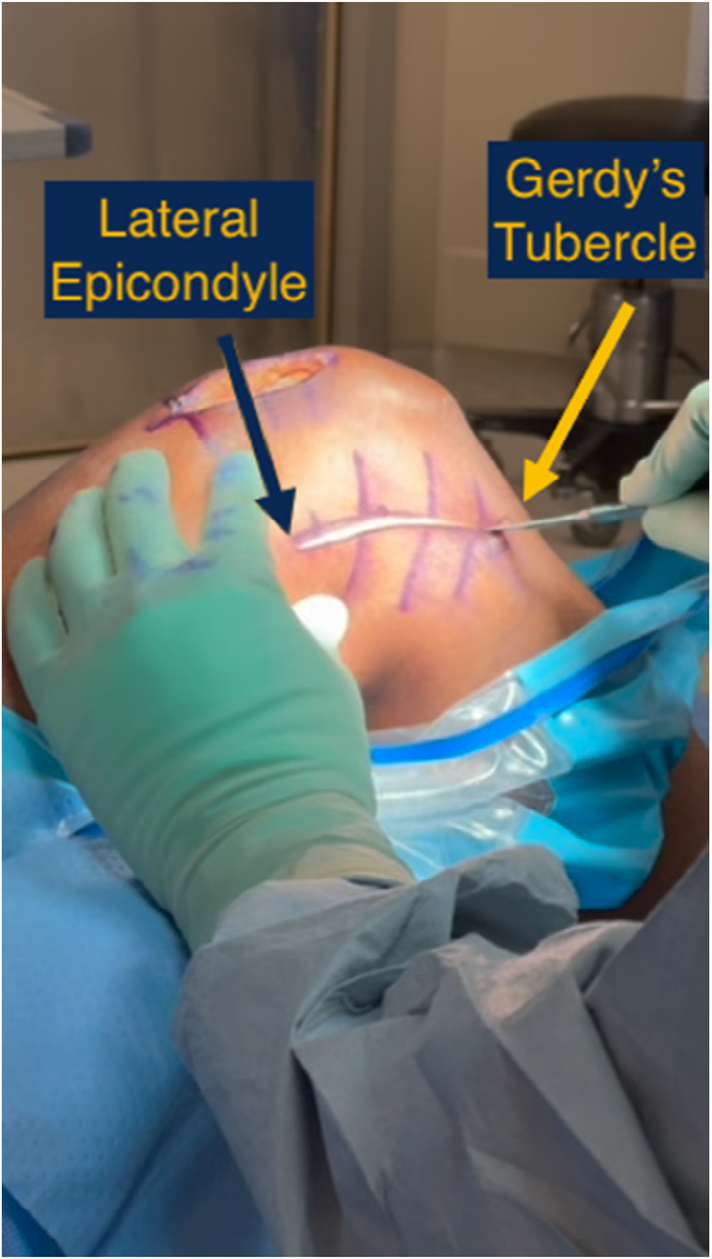
The right knee is shown in supine position with the knee flexed to approximately 90°. Identification of surface anatomy and lateral incision for LET. With the patient supine and the operative knee flexed, surface landmarks including the lateral epicondyle, Gerdy's tubercle, and the course of the IT band are marked. A longitudinal lateral incision is made centered over the lateral epicondyle and extending distally toward Gerdy's tubercle, stopping short of the tubercle to preserve the distal IT band attachment. This exposure provides access for IT band graft harvest and subsequent femoral LET fixation while minimizing soft‐tissue disruption. (IT, iliotibial; LET, lateral extra‐articular tenodesis.)

**FIGURE 3:**
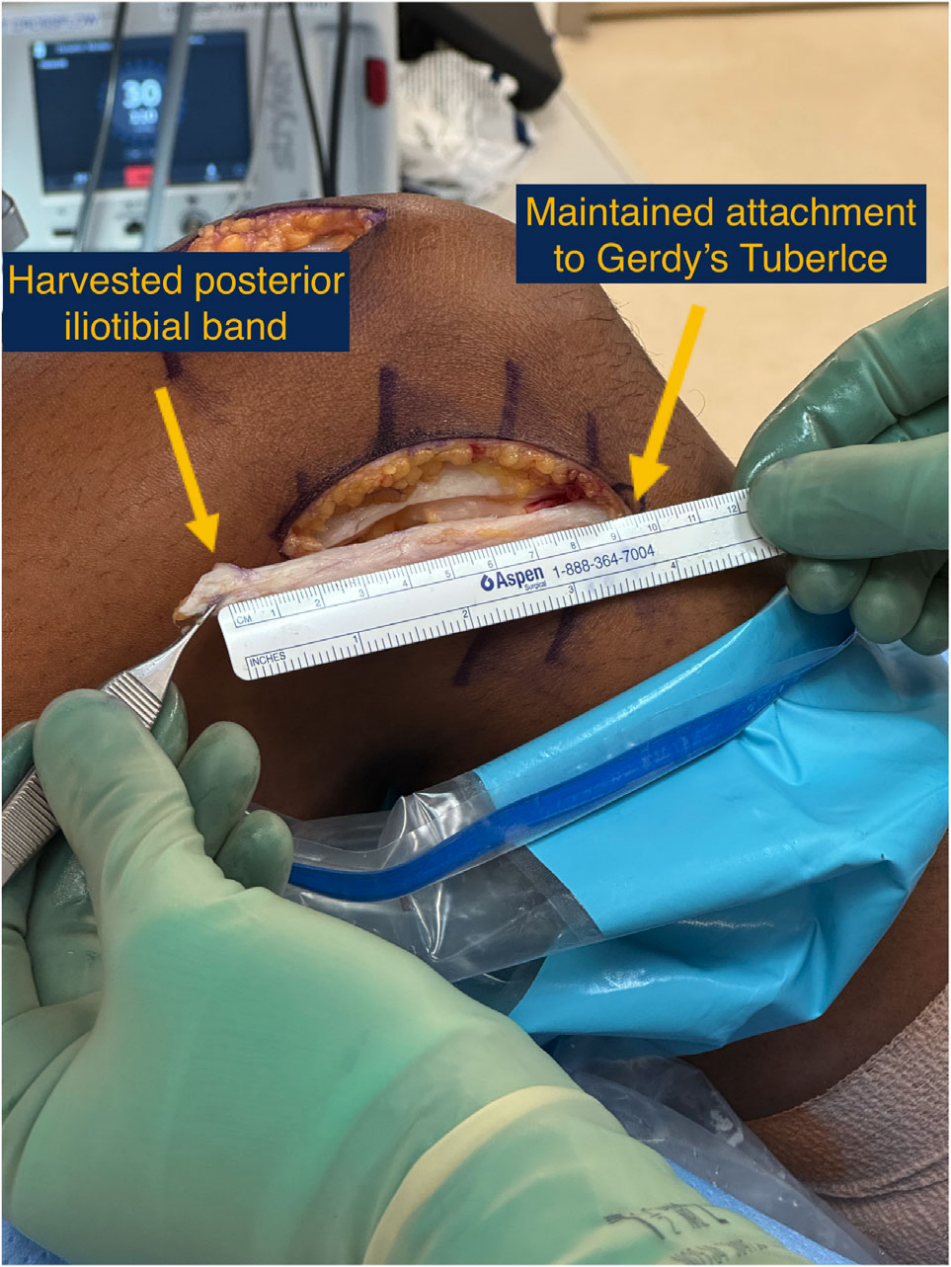
The right knee is shown in supine position with the knee flexed to approximately 90°. Harvest of the posterior IT band graft for LET. Through the lateral incision, a strip of the posterior third of the IT band has been sharply harvested, maintaining its distal attachment to Gerdy's tubercle. The graft has been elevated from the underlying tissues but has not yet undergone proximal preparation or whipstitching. Preservation of the distal attachment maintains native length and orientation for subsequent passage deep to the lateral collateral ligament and femoral fixation. (IT, iliotibial; LET, lateral extra‐articular tenodesis.)

**FIGURE 4:**
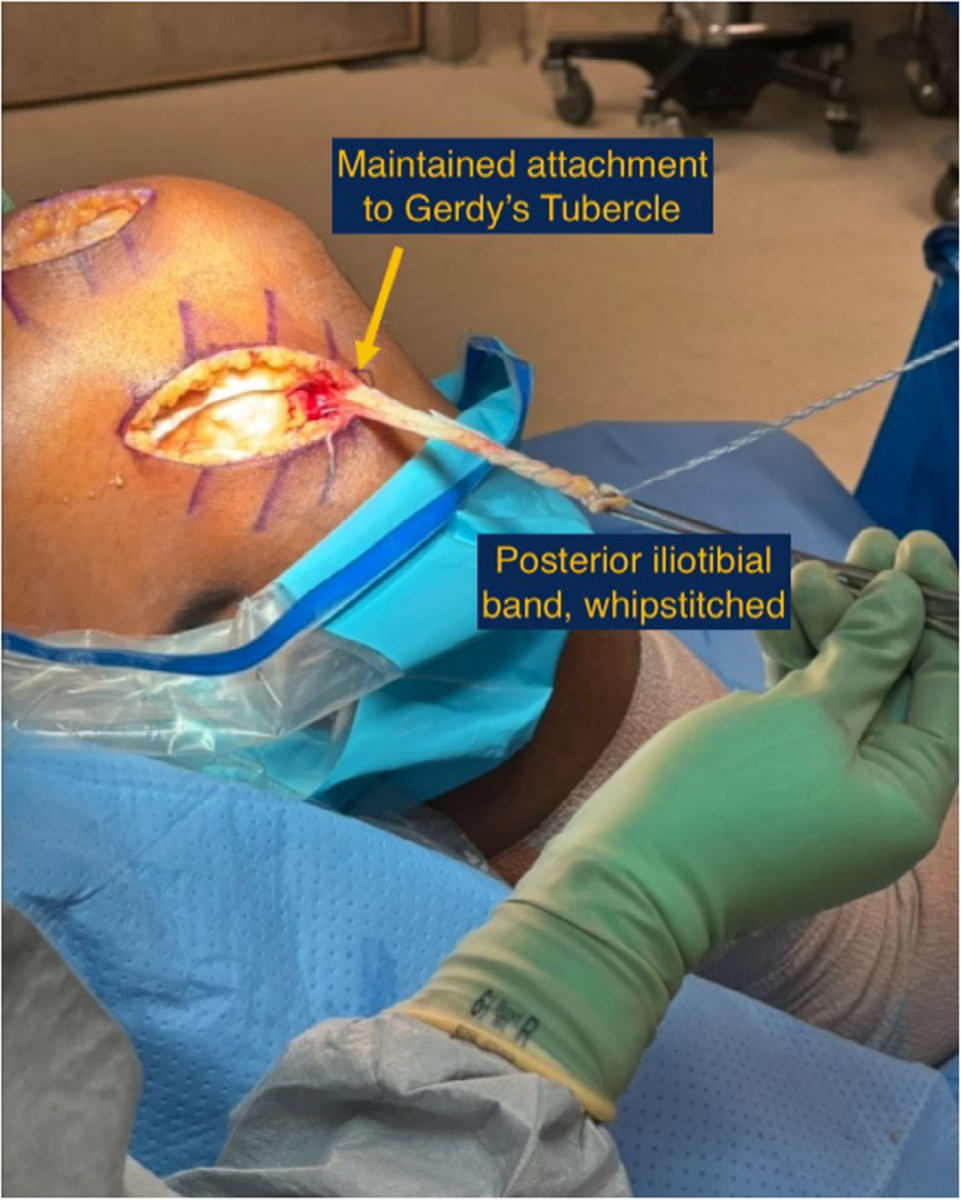
The right knee is shown in supine position with the knee flexed to approximately 90°. Preparation of the posterior IT band graft for LET. Following harvest, the proximal end of the posterior IT band graft is prepared with a nonabsorbable whipstitch while maintaining its distal attachment to Gerdy's tubercle. The graft is sized to ensure sufficient length for passage deep to the lateral collateral ligament, femoral fixation, and subsequent doubling back for onlay reinforcement. Proper graft preparation facilitates controlled tensioning and secure fixation during the LET portion of the procedure. (IT, iliotibial; LET, lateral extra‐articular tenodesis.)

**FIGURE 5:**
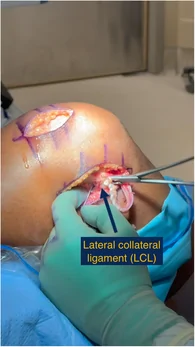
The right knee is shown in supine position with the knee flexed to approximately 90°. Passage of the LET graft deep to the LCL. With the patient supine and the operative knee flexed, a right‐angle clamp is passed deep to the LCL through the lateral incision. The prepared posterior IT band graft is then shuttled deep to the LCL in an anterograde fashion using passing sutures. (IT, iliotibial; LCL, lateral collateral ligament; LET, lateral extra‐articular tenodesis.)

**FIGURE 6:**
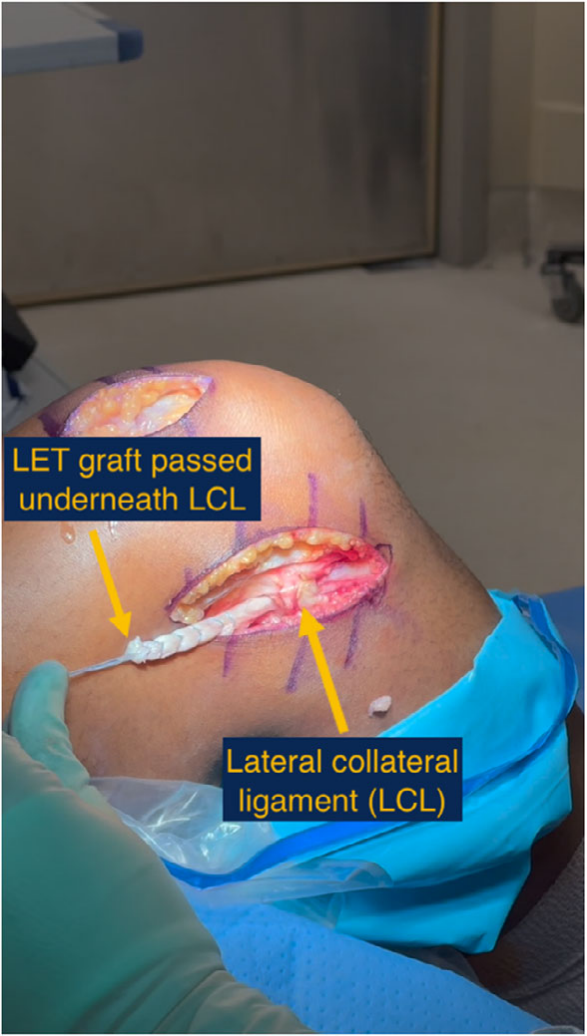
The right knee is shown in supine position with the knee flexed to approximately 90°. Final positioning of the LET graft after passage deep to the LCL. The posterior IT band graft is shown fully passed and lying deep to the LCL, with the distal attachment preserved at Gerdy's tubercle and the proximal sutured end positioned for subsequent femoral fixation. (IT, iliotibial; LCL, lateral collateral ligament; LET, lateral extra‐articular tenodesis.)

Prior to graft harvest for the ACLR, a sharp 7 cm incision is made centered on the lateral epicondyle and extending toward Gerdy's tubercle, stopping 1 cm short of the tubercle proper (Figure 2). Following dissection through subcutaneous tissue, the iliotibial band is identified and cleared of residual soft tissue using a large key elevator. The posterior third of the iliotibial band is marked, and a 1 cm wide strip measuring at least 8 cm is sharply made using a 15 blade, taking care to maintain the distal attachment to Gerdy's tubercle and avoid iatrogenic damage to the underlying lateral collateral ligament (LCL) (Figure 3). The proximal aspect is then whipstitched using a #2 suture (Figure 4). A vertical incision is made on either side of the LCL through the overlying biceps bursa using electrocautery, and a right‐angle clamp is passed deep to the LCL in an anterograde fashion (Figure 5). The clamp is spread to improve graft passage, and the passing sutures are used to pass the LET graft deep to the LCL (Figure 6). Once this is completed, the lateral incision is packed with a lap sponge and covered with Ioban. Graft harvest and preparation for ACLR then proceeds according to the surgeon's preferred technique.

A standard anterolateral portal is created on the front of a knee using an 11‐blade, and an anteromedial portal is localized using an 18‐gauge spinal needle. Diagnostic arthroscopy of the patellofemoral, medial, and lateral compartments is performed while viewing through the anterolateral portal. Intra‐articular work for ACLR preparation is completed, as well as any additional work pertaining to meniscus or chondral pathology as necessary. A flexible guide pin is then introduced via the anteromedial portal through a femoral aiming guide, and the knee is held flexed to 120° by a surgical assistant. The guide pin is driven through the bone and out the lateral skin and is then clamped with a Kocher clamp. The proper size flexible reamer corresponding to the measured graft diameter is placed over the guidewire and is used to create a 25 mm long tunnel.

**FIGURE 7:**
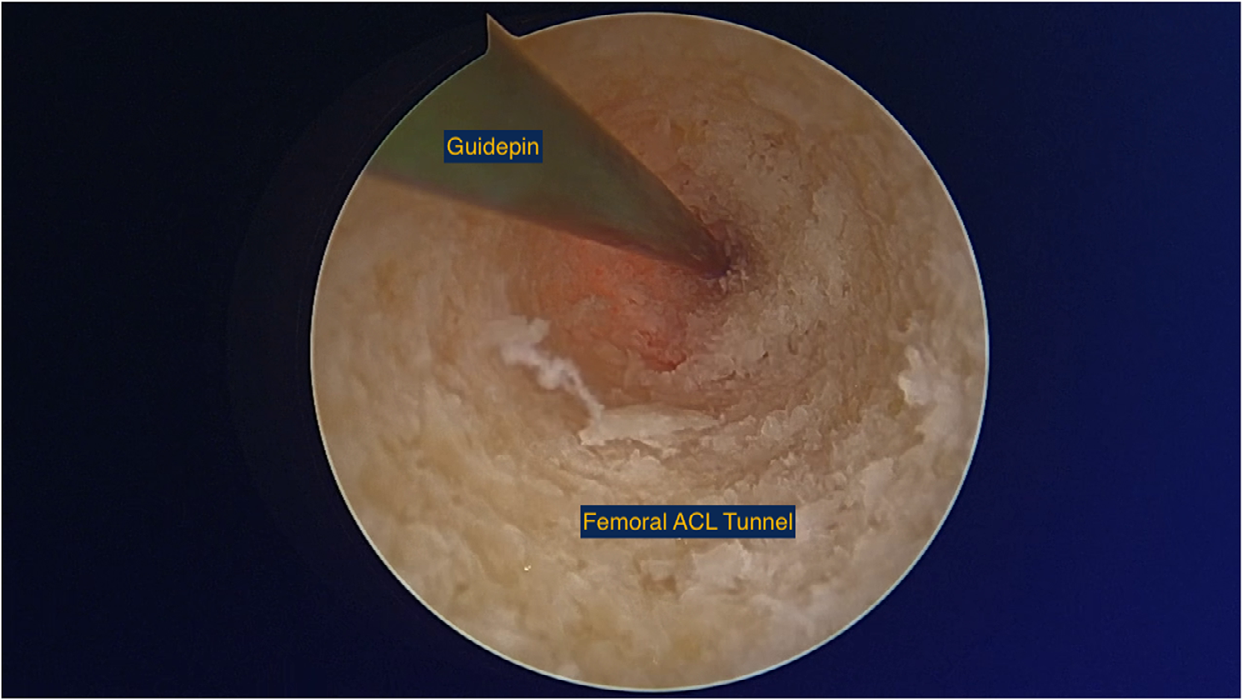
Arthroscopic image of the right knee viewed through the anteromedial portal with the patient supine and the knee flexed to 90°. Arthroscopic visualization of the femoral ACL tunnel with the flexible guide pin in place. With the patient supine and the operative knee flexed at 90°, the arthroscope is placed through the anteromedial portal to visualize the femoral tunnel during LET anchor drilling. Direct intra‐articular visualization at this stage allows immediate identification of potential tunnel convergence, while the guide pin allows immediate tactile feedback of potential tunnel convergence additionally. (ACL, anterior cruciate ligament; LET, lateral extra‐articular tenodesis.)

**FIGURE 8:**
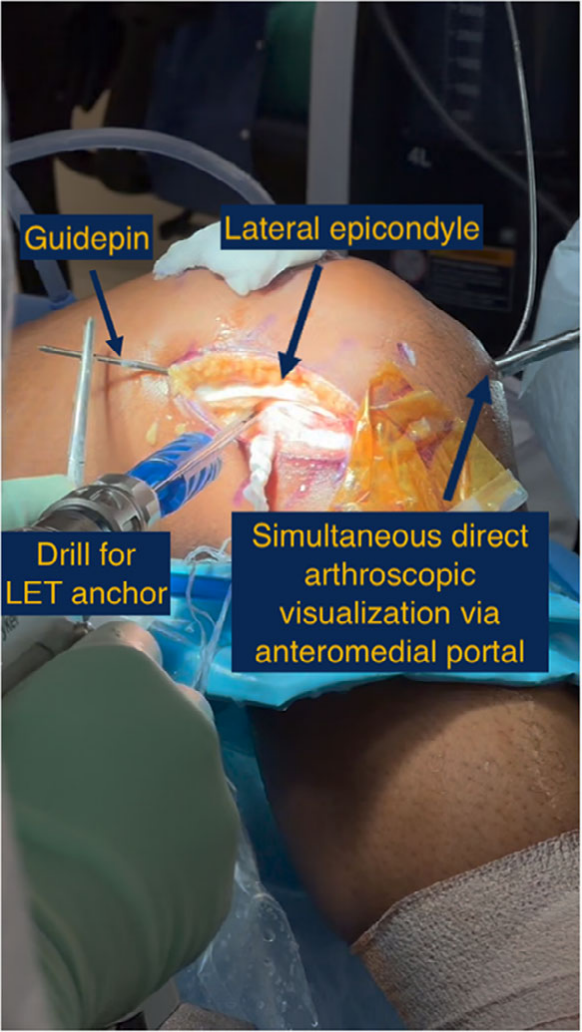
The right knee is shown in supine position with the knee flexed to approximately 90°. Arthroscopic visualization is performed through the anteromedial portal. Femoral anchor placement for LET performed with direct arthroscopic visualization of femoral ACL tunnel. With the flexible guide pin maintained within the femoral ACL tunnel and the arthroscope viewing through the anteromedial portal, the LET pilot hole is drilled through the lateral incision at the femoral fixation site proximal and posterior to the lateral epicondyle, aiming drill trajectory both anteriorly and proximally. Continuous intra‐articular visualization confirms a safe drill trajectory without femoral tunnel breach, allowing secure anchor deployment while avoiding damage to the ACL graft or suspensory fixation device. (ACL, anterior cruciate ligament; LET, lateral extra‐articular tenodesis.)

**FIGURE 9:**
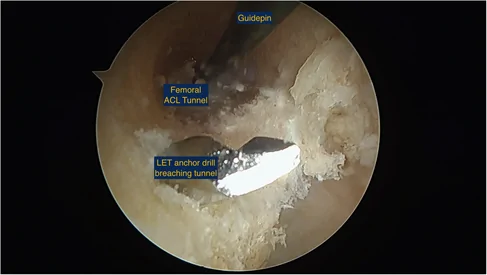
Arthroscopic image of the right knee viewed through the anteromedial portal with the patient supine and the knee flexed to 90°. Arthroscopic visualization of femoral ACL tunnel showing convergence of the LET anchor drill tunnel trajectory. With the flexible guide pin retained within the femoral tunnel and the arthroscope viewing through the anteromedial portal, the tip of the LET pilot drill is visualized breaching the femoral tunnel. This intra‐articular view, combined with tactile feedback from contact between the drill and the flexible guide pin, alerts the surgeon to impending tunnel convergence and allows immediate redirection of the drill to avoid damage to the ACL graft or suspensory fixation device. (ACL, anterior cruciate ligament; LET, lateral extra‐articular tenodesis.)

The surgical assistant then releases the knee flexion to 90°, and the arthroscope is inserted into the anteromedial portal to visualize the femoral tunnel and guidewire (Figure 7). While continuing to visualize the joint, attention is turned to the lateral incision for placement of the LET anchor. A point just proximal and posterior to the lateral epicondyle is chosen as the site of the anchor for a modified Lemaire LET. The pilot hole for the anchor is drilled through the guide, and the femoral tunnel is simultaneously visualized for drill penetration (Figure 8). If the drill tip is seen breaching the tunnel (Figure 9), or if resistance is felt as the drill contacts the flexible guide pin, the drill is then removed and redirected until a proper drill trajectory is obtained. The drill is then removed, and the anchor is placed and set against the lateral cortex per the manufacturer's specifications.

**FIGURE 10:**
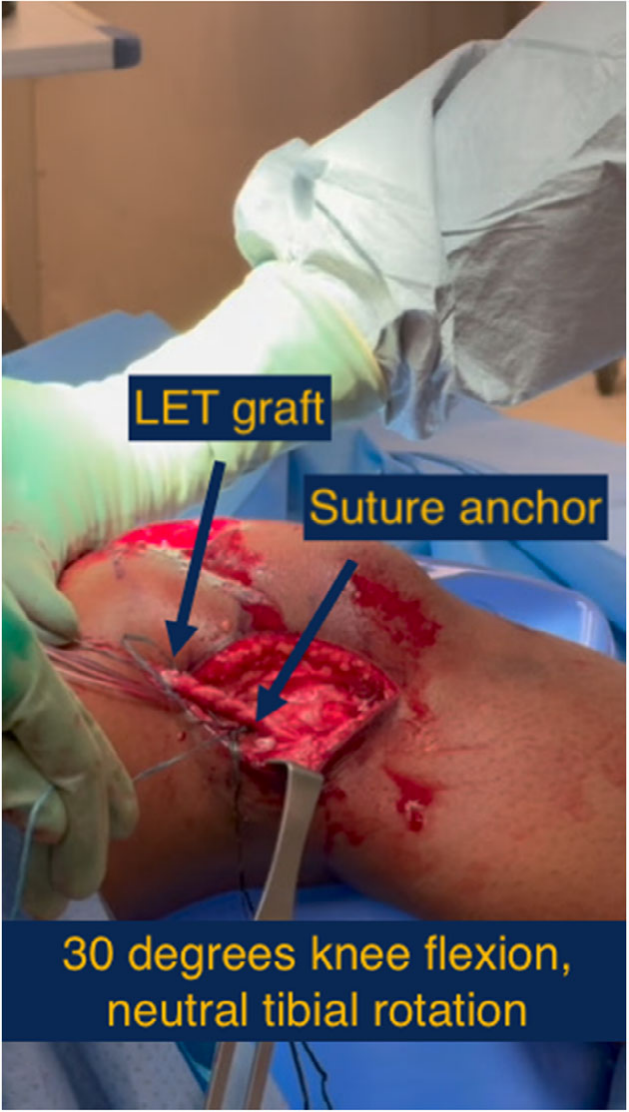
The right knee is shown in supine position with the knee flexed to approximately 30° and the tibia in neutral rotation. Final tensioning and fixation of the LET graft. With the patient supine and the operative knee positioned in approximately 30° of flexion and neutral rotation, the LET graft is tensioned proximally and secured using the knotless femoral anchor in an onlay fashion. The graft is then doubled back over itself and reinforced with additional fixation sutures. (LET, lateral extra‐articular tenodesis.)

The ACLR then proceeds with drilling of the tibial tunnel, suture passage, graft passage, and graft fixation; these steps may be completed according to the surgeon's preferred technique. After the graft has been secured, the LET graft is passed through the knotless loop of the anchor. The knee is then placed in approximately 30° of flexion and neutral rotation, and the knotless loop is tightened down while pulling tension proximally on the LET graft sutures to secure the graft in an onlay fashion (Figure 10). The graft is then doubled back over itself and secured by passing the remaining sliding suture within the anchor through the graft ends in a mattress fashion. The graft sutures are cut, and the iliotibial band is then closed using a running barbed suture.

## DISCUSSION

Although previous studies have focused on adjusting LET tunnel/anchor trajectory to avoid convergence with the ACL graft,^4^, ^5^, ^12^, ^13^ our technique uses a flexible guide pin, a modified drilling sequence, and a 2.6 mm knotless all‐suture anchor to safely and efficiently perform a modified Lemaire LET in conjunction with ACLR using suspensory graft fixation. Key advantages include combined visual and tactile feedback during LET anchor placement, allowing for immediate correction if necessary, and avoidance of graft suspensory fixation by utilizing early anchor placement. Additionally, the use of a flexible guide pin avoids the need for maintaining knee hyperflexion during LET anchor placement.

Recent literature has shown that tunnel convergence remains a significant concern when performing LET in conjunction with ACLR. A study assessing postoperative computed tomography in 52 patients who underwent ACLR with LET showed a convergence rate of 15.4%, with 26.9% having less than 5 mm between tunnels.^14^ In a cadaveric study aimed at estimating the risk based on LET technique, tunnel convergence was found to be as high as 70%.^4^ Furthermore, recent studies have shown a high level of variability in LET femoral fixation point, with only 53% located in the desired isometric zone despite the use of palpable anatomic landmarks.^15^ This suggests that relying on these landmarks and tunnel trajectory alone may not be sufficient to reliably achieve safe tunnel placement that avoids convergence.

Although ALLR serves as an alternative to LET, this technique requires additional graft and fixation devices and also requires 2 tunnels, carrying a similarly high risk of tunnel convergence. In a recent cross‐sectional study of 227 patients who underwent ACLR with ALLR, postoperative computed tomography showed a tunnel convergence rate of 53.3%,^13^ whereas in a cadaveric study, convergence rate was as high as 67%.^5^ Additionally, ALLR introduces additional donor site morbidity when an autograft is used, and its long‐term benefits over LET remain debated.

Despite the advantages of our technique, certain disadvantages and potential pitfalls must be considered. Although intraoperative visualization helps mitigate tunnel convergence, the normal flow of ACLR is disrupted by requiring transition from intra‐articular to extra‐articular work and specialized guides and flexible reamers are required. Improper LET anchor placement may lead to poor graft isometry and early failure,^6^ and if deployed within the femoral tunnel, this may prevent ACL graft seating.

This technique provides a reliable and reproducible method for ACLR with LET, reducing the risk of tunnel convergence and graft damage while preserving the benefits of suspensory femoral fixation. By optimizing the sequencing tunnel drilling and anchor placement, this approach enhances surgical efficiency and safety, ultimately improving patient outcomes.

## DISCLOSURES

The author (C.A.L.) declares the following financial interests/personal relationships which may be considered as potential competing interests: C.A.L. is a paid consultant for CONMED Linvatec, Johnson & Johnson, and Moximed, outside the submitted work. The other authors (M.F., S.T., A.V., R.D.F.) declare that they have no known competing financial interests or personal relationships that could have appeared to influence the work reported in this paper.
